# The Anti-Inflammatory Effects of the Methanolic Extract and Fractions from *Davilla elliptica* St. Hil. (Dilleniaceae) on *Bothrops jararaca* Envenomation

**DOI:** 10.3390/ijms160612454

**Published:** 2015-06-02

**Authors:** Catarine Massucato Nishijima, Flavia Karina Delella, Clenilson Martins Rodrigues, Daniel Rinaldo, Monica Valdyrce dos Anjos Lopes-Ferreira, Lucia Regina Machado da Rocha, Wagner Vilegas, Sergio Luis Felisbino, Clélia Akiko Hiruma-Lima

**Affiliations:** 1 Departamento de Fisiologia, Instituto de Biociências, Universidade Estadual Paulista-UNESP, CEP 18618-970 Botucatu, São Paulo, Brazil; E-Mails: cat.nishi@ig.com.br (C.M.N.); lrocha@ibb.unesp.br (L.R.M.R.); 2 Departamento de Morfologia, Instituto de Biociências, Universidade Estadual Paulista-UNESP, CEP 18618-970 Botucatu, São Paulo, Brazil; E-Mails: fdelella@ibb.unesp.br (F.K.D.); felisbin@ibb.unesp.br (S.L.F.); 3 Centro Nacional de Pesquisa de Agroenergia (CNPAE), Empresa Brasileira de Pesquisa Agropecuária, CEP 70770-901, Brasília, Brazil; E-Mail: clenilson.rodrigues@embrapa.br; 4 Faculdade de Ciências de Bauru, Universidade Estadual Paulista-UNESP, CEP 17033-360 Bauru, Brazil; E-Mail: danielrinaldo@fc.unesp.br; 5 Laboratório Especial de Toxinologia Aplicada. Butantan, Instituto Butantan, CEP 05503-900 São Paulo, Brazil; E-Mail: mlferrei@usp.br; 6 Campus Experimental do Litoral Paulista, Universidade Estadual Paulista-UNESP, CEP 11330-900 São Vicente, São Paulo, Brazil; E-Mail: vilegas@gmail.com

**Keywords:** *Davilla elliptica* St. Hil, anti-inflammatory effect, MMP-9, MMP-2, *Bothrops jararaca* venom

## Abstract

Inflammation and haemorrhage are the main characteristics of tissue injury in botropic envenomation. Although some studies have shown that anti-venom prevents systemic reactions, it is not efficient in preventing tissue injury at the site of the bite. Therefore, this work was undertaken to investigate the anti-inflammatory effects of the methanolic extract and fractions from *D. elliptica* and to evaluate the role of matrix metalloproteinases (MMPs) in this process. Effects of the extract and fractions from *D. elliptica* were evaluated using a carrageenan-induced paw oedema model in rats, and leukocyte rolling was visualized by intravital. The quantification of MMPs activities (MMP-2 and MMP-9) extracted from the dermis of mice treated with extract and fractions alone or incubated with venom was determined by zymographic analyses. Our results show that intraperitoneal (i.p.) injection of fractions significantly reduced paw oedema after the carrageenan challenge. Treatment with the tannins fraction also resulted in considerable inhibition of the rolling of leukocytes and this fraction was able to decrease the activation of MMP-9. These results confirmed the anti-inflammatory activity of the methanolic extract and tannins fraction of *D. elliptica* and showed that the dermonecrosis properties of *B. jararaca* venom might be mediated through the inhibition of MMP-9 activity.

## 1. Introduction

Inflammatory reaction and haemorrhage are the main characteristics of tissue injury in botropic envenomation [1]. Haemorrhage and oedema are consequences of proteolytic action promoted by venom that abolishes the vascular integrity, induces necrosis, promotes the activation of fibroblasts, and stimulates cell recruitment of mononuclear phagocytes and granulocytes to the site of the bite [2]. All of these reactions are triggered by venom proteins belonging to a Zn^2+^-dependent enzyme family known as snake venom metalloproteinases (SVMP) [3]. In addition to the proteolytic effects of SVMPs on the extracellular matrix, these effects are also potentiated by endogenous matrix metalloproteinases (MMP) activities. MMPs play a role in the remodelling and homeostasis of extracellular compounds, and they promote the migration of leukocytes from vessels to the tissue during the inflammatory process via the degradation of extracellular matrix components, such as laminins, collagens and proteoglycans [4].

Although some studies have shown that anti-venom prevents systemic reactions, it is not efficient in preventing tissue injury at the site of the bite [5]. Thus, in addition to the anti-inflammatory effect, substances that inhibit MMP activities may be important targets in the control of local and systemic effects of bothropic envenomation [6]. Moreover, the search for novel MMP inhibitors will provide useful tools in the treatment of other conditions that involve inflammation and MMP activity, such as arthritis, asthma, periodontitis, cardiovascular diseases and cancer [7].

Many medicinal plants have reported activities against snakebites [8]. These plants contain secondary metabolites, such as triterpenoids, flavonoids and coumarins that inhibit MMPs and phospholipases from ophidic venoms [9].

*Davilla elliptica* St. Hil. (Dilleniaceae), a common Brazilian savanna species, is employed in popular medicine in a tea form to treat ulcers, diarrhoea, inflammation and gastric pain [10]. The phytochemical profile of the methanolic extract from *Davilla elliptica* leaves demonstrated the presence of phenolic acid derivatives, condensed tannins and acylglycoflavonoids [11]. The potential of this medicinal species was evaluated by Azevedo and colleagues [12] in which the authors described the antinociceptive effect of the hydroalcoholic extract of the stems from *Davilla elliptica.* Campos and colleagues [13] isolated myricetin-3-*O*-β-galactopyranoside from the leaves of this species, which contributes to the antinociceptive effect from this medicinal plant. Nishijima and colleagues [14] showed that the methanolic extract and rich flavonoids fraction from *Davilla elliptica* had total venom neutralization capacity against the haemorrhagic activity of *B. jararaca*. Thus, the aims of the present study were to evaluate the anti-inflammatory action of the methanolic extract and the enriched fractions containing flavonoids and tannins from *D. elliptica*, as well as to characterize the effect of these fractions on MMP activities against *Bothrops jararaca* venom inoculation.

## 2. Results and Discussion

Our previous study showed that the methanolic extract of *Davilla elliptica* (125, 250 or 500 mg/kg) given via the oral route failed to reduce paw oedema after the carrageenan test [15]. This effect was probably due to the bioavailability of the extract with tannins in their composition because in evaluating the pharmacological effect *in vivo*, polymeric proanthocyanidins did not cross the intestinal wall barrier and were not digested in the stomach. Polymeric proanthocyanidins are known to have depressing effects on protein digestibility due to their ability to bind and precipitate proteins [16,17]. These factors probably affected the anti-oedematogenic evaluation of this extract administered via the oral route; however, as shown in Table 1, when we administered the extract of *Davilla elliptica* via the intraperitoneal route (15.62 and 31.25 mg/kg), oedema was considerably reduced in the first, second, third and fourth hour after the carrageenan administration (*p* < 0.01), and no significant differences were observed between these doses, although the lower dose of this extract (7.81 mg/kg) only inhibited paw oedema at the third hour after administration.

**Table 1:** Effects of administration of methanolic extract from *Davilla elliptica* leaves via the intraperitoneal route on carrageenan-induced rat paw oedema.

Treatment	Dose (mg/kg)	1st h	2nd h	3rd h	4th h
Vehicle	–	0.93 ± 0.19	1.65 ± 0.30	2.61 ± 0.21	2.83 ± 0.20
Piroxicam	30	0.29 ± 0.11 **	0.26 ± 0.04 **	0.83 ± 0.14 **	1.37 ± 0.24 *
Methanolic extract	7.81	0.71 ± 0.12	0.83 ± 0.26	1.07 ± 0.25 *	1.92 ± 0.13
	15.62	0.30 ± 0.06 **	0.40 ± 0.07 **	0.88 ± 0.15 **	1.76 ± 0.21 **
	31.25	0.38 ± 0.01 **	0.50 ± 0.12 **	0.59 ± 0.12 **	1.01 ± 0.27 *

Data are means ± S.E.M. of oedema volume (mL) (*n* = 5–6). ANOVA, followed by Dunnett’s test. * *p* < 0.05, ** *p* < 0.01 *vs.* the saline group within the same period of observation.

Thus, the subsequent evaluations were carried out at a dose of 15.62 mg/kg of extract and also with both fractions (flavonoid and tannin) to analyse comparative effects between them. The ability of the extract to inhibit paw oedema was abolished when the flavonoids fraction was evaluated (Table 2). The fractionation from the methanolic extract of *D. elliptica* showed that only the tannins fraction presented an anti-oedematogenic effect at all observed hours (Table 3).

**Table 2:** Effects of administration of flavonoid fraction from *Davilla elliptica* leaves via the intraperitoneal route on carrageenan-induced rat paw oedema.

Treatment	Dose (mg/kg)	1st h	2nd h	3rd h	4th h
Vehicle	–	0.53 ± 0.09	1.18 ± 0.20	1.92 ± 0.18	2.43 ± 0.20
Piroxicam	30	0.25 ± 0.05 *	0.32 ± 0.05 **	0.71± 0.13 **	1.06 ± 0.19 **
Flavonoids fraction	15.62	0.46 ± 0.06	0.73 ± 0.13	1.53 ± 0.17	2.20 ± 0.19

Data are means ± S.E.M of oedema volume (mL) (*n* = 5–6). ANOVA, followed by Dunnett’s test. * *p* < 0.05, ** *p* < 0.01 *vs.* the saline group within the same period of observation.

**Table 3:** Effects of administration of tannins fraction from *Davilla elliptica* leaves via the intraperitoneal route on carrageenan-induced rat paw oedema.

Treatment	Dose (mg/kg)	1st h	2nd h	3rd h	4th h
Vehicle	–	0.55 ± 0.06	1.63 ± 0.24	2.71 ± 0.25	2.79 ± 0.22
Piroxicam	30	0.38 ± 0.08	0.49 ± 0.09 **	0.97 ± 0.08 **	1.12 ± 0.18 **
Tannins fraction	15.62	0.30 ± 0.04 *	0.79 ± 0.06 *	1.81 ± 0.20 **	2.75 ± 0.17

Data are means ± S.E.M (*n* = 5–6) of oedema volume (mL). ANOVA, followed by Dunnett’s test. * *p* < 0.05, ** *p* < 0.01 *vs.* the saline group within the same period of observation.

Our positive control group, given piroxicam (30 mg/kg), had significantly inhibited paw oedema after carrageenan treatment during all times of observation, contributing to the validation of this assay. Thus, the methanolic extract and the fraction of tannins from *D. elliptica* showed anti-oedematogenic effects during this phase (characterized by the first 4 h of inflammation in the model of paw oedema induced by carrageenan) that were related to the release of bradykinin, histamine, serotonin and prostaglandins. These results could be related to condensed tannins, which are the major secondary metabolites of the methanol extract from the leaves of *D. elliptica*. The phytochemical profile revealed the presence of 41.2% condensed tannins and 5.1% total flavonoids [15]. Proanthocyanidins, also called condensed tannins, are known for their anti-inflammatory effects, probably related to the ability of tannins to inhibit enzymes. Such inhibition would occur through the formation of complexes with proteins for the binding of the proanthocyanidins, including phenolic residues and polar groups of enzymes [18].

**Figure 1:**
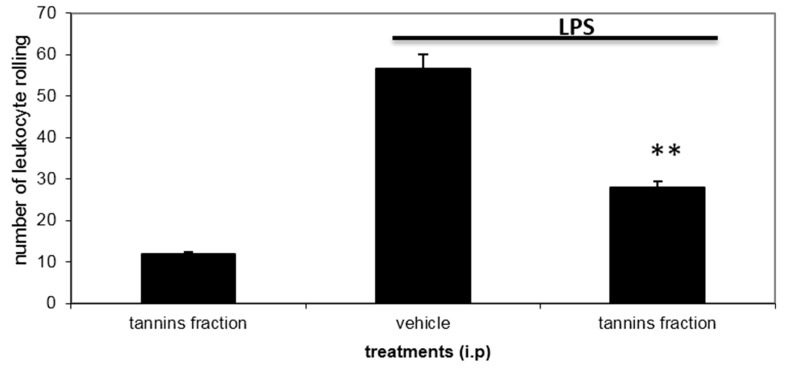
Effect of the tannins’ fraction from *D. elliptica* on leukocyte rolling number after lipopolysaccharides (LPS) topical application to the cremaster muscle in mice (intravital microscopy).Data are means ± S.E.M (*n* = 11). ANOVA, followed by Dunnett’s test. ** *p* < 0.01 *vs.* the saline group within the same period of observation.

Inflammation is the main characteristic of envenomation by the *Bothrops* genus, and after tissue injury, a variety of inflammatory mediators, such as vasoactive amines and eicosanoids are produced by endothelial cells and resident cells, especially mast cells, macrophages and dendritic cells near the injured site [19]. These mediators lead all of the inflammatory events in response to injury. Moreover, circulating leukocytes, initially neutrophils, migrate to the tissue under the influence of vasodilatation and chemotactic agents [20,21]. This process involves the rolling and adhesion of leukocytes in the microvascular endothelium, followed by transmigration through the vessel wall to the extravascular tissue. Leukocyte rolling was the important parameter for evaluating the anti-inflammatory effect of the tannins’ fraction of *D. elliptica* (15.62 mg/kg, i.p.)*.* Using intravital microscopy, our results showed (Figure 1) that the tannins’ fraction caused a significant reduction in leukocyte rolling (50%) in the post-capillary venules of the cremaster muscle of mice induced by the topical application of lipopolysaccharides (LPS) (*p* < 0.01).

Macrophages, mast cells, lymphocytes, fibroblasts and neutrophils are the main cellular sources of MMPs inducible under inflammatory conditions. Among them, MMP-9 (a gelatinase B) degrades collagen IV and V, fibronectin and elastin. It is synthesized in the form of latent zymogen (92 kDa) and is then released and cleaved in active form (81 kDa). MMP-9 has been associated with the initiation and end of the inflammatory response, the transmigration of leukocytes, the regulation of IL-1β and IL-8 and the process of tumour invasion. MMP-9 expression is regulated at the transcriptional level by hormones, growth factors and chemokines, while MMP-2 (a gelatinase type A) is expressed constitutively by healthy tissues and is involved in normal tissue remodelling. These MMP activities are also regulated by the activation of zymogen and by 1:1 stoichiometric interaction with tissue inhibitors of MMPs (TIMPs) [22,23]. Rucavado and colleagues [6] demonstrated the increased activity of MMP-9 in the gastrocnemius muscle of mice after injection of BAP1, a metalloproteinase isolated from *Bothrops asper*. The activation of pro MMP-2 in fibroblasts after incubation with *Bothrops asper* venom was observed by Saravia-Otten and colleagues [24], however, there are no reports regarding MMP-2 and MMP-9 activities induced by *Bothrops jararaca* in animal models.

**Figure 2:**
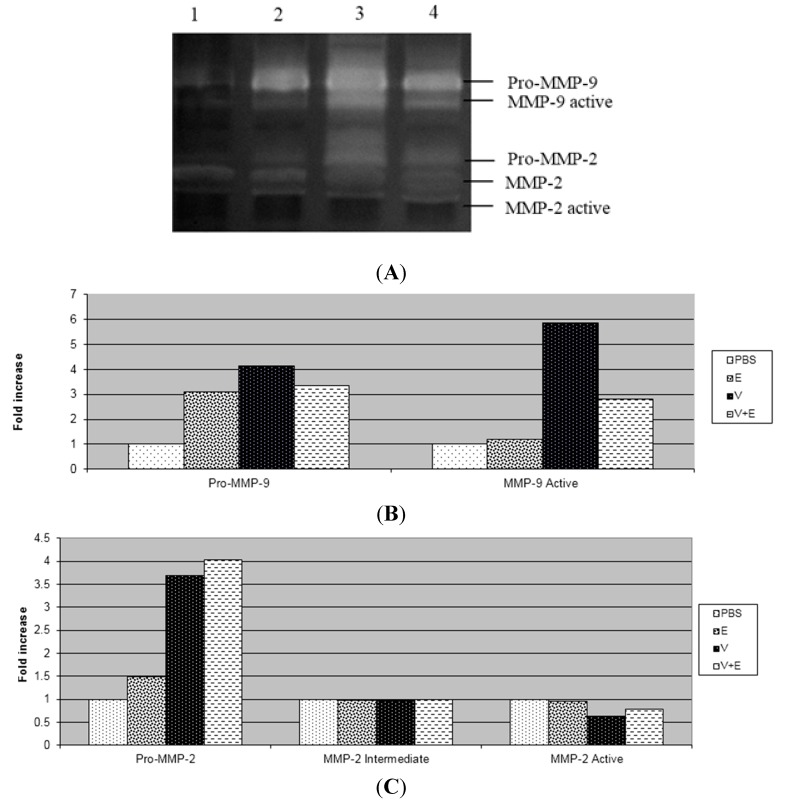
Effect of the methanolic extract from *Davilla elliptica* on the gelatinolytic activity of MMP-9 and MMP-2 in mouse dermis. (**A**) Gelatinolytic activity of MMP-2 and MMP-9: PBS (lane 1); Methanolic extract (E) (lane 2); venom (V) (lane 3); venom + methanolic extract (V + E) (lane 4); (**B**) The gelatinolytic activity of MMP-9 by integrated optical density (IOD); (**C**) The gelatinolytic activity of MMP-2 by integrated optical density (IOD). The results are presented as fold increases.

**Figure 3:**
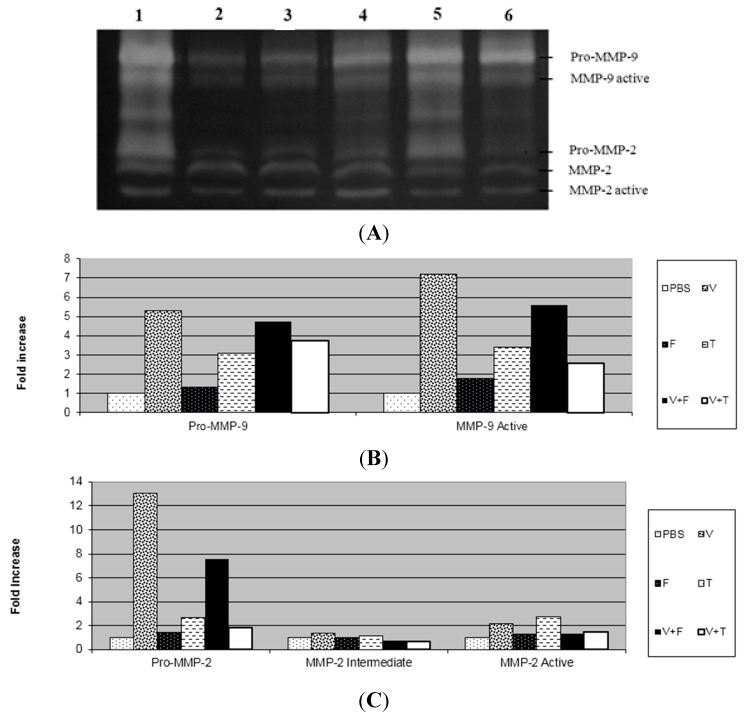
Effect of the tannin and flavonoid fractions from *Davilla elliptica* on the gelatinolytic activity of MMP-2 and MMP-9 in mouse dermis. (**A**) The gelatinolytic activity of MMP-2 and MMP-9: Venom (V) (lane 1); PBS (lane 2); Flavonoids fraction (F) (lane 3); Tannins fraction (T) (lane 4); Venom + flavonoids fraction (V + F) (lane 5); Venom + tannins fraction (V + T) (lane 6); (**B**) The gelatinolytic activity of MMP-9 by integrated optical density (IOD); (**C**) The gelatinolytic activity of MMP-2 by integrated optical density (IOD). The results are presented as fold increases.

In this study, after three days of application of *B. jararaca* venom into the dermis, we observed through the zymography technique that the venom increased the activity of pro-MMP-2, active-MMP-2, pro-MMP-9 and active-MMP-9 and there was no change in the activity of intermediate MMP-2 in relation to the PBS group (Figure 2 and Figure 3). The extract and tannins fraction were able to decrease the activation of MMP-9 induced by the venom, but the same result was not observed with the flavonoids fraction from *D. elliptica*. The tannins fraction also decreased the activity of pro-MMP-2 in relation to the venom. Similar results were obtained in relation to paw oedema, as we found that only the extract and tannins fraction decreased the oedema induced by carrageenan.

The absence of necrosis in the dermis of animals that received the mixture of venom with the extract or tannins fraction was due to the decreased activity of MMP-9 caused by the extract (Figure 2) and tannins fraction (Figure 3). These results suggest that the active MMP-9 form is closely related to necrosis and the inflammatory process induced by *Bothrops jararaca* venom. Although the flavonoids fraction from *D. elliptica* was not able to inhibit the inflammatory process nor to alter the activity of MMPs, it has the ability to neutralize the haemorrhagic process when incubated with the venom of *Bothrops jararaca*, probably by the chelating property of phenolic compounds [14].

These results suggest that condensed tannins are responsible for the anti-inflammatory effect of this vegetal species by reducing paw oedema and the rolling of leukocytes, as well as by decreasing the activity of MMP-9 during the inflammatory process caused by *Bothrops jararaca*. Several drugs have been developed to block the synthesis of MMPs and prevent the dermonecrosis process. Some of the drugs used to treat dermonecrosis after poisoning are inhibitors of the infiltration of polymorphonuclear cells [25]. The effect of condensed tannins on MMPs has been already reported. Oligomeric procyanidins isolated from fruits of *Chaenomeles japonica* decreased the activity of MMP-2 and MMP-9 in the culture medium of mononuclear cells from human blood. Bawadi and colleagues [26] showed the inhibition of the production of MMP-2 and MMP-9 by tannins of the aqueous fraction of black beans in a medium of tumour cells of the breast, colon and prostate.

## 3. Experimental Section

### 3.1. Drugs and Venom

Piroxicam was purchased from Pfizer (São Paulo, Brazil), carrageenan was purchased from Sigma Chemical Co. (St. Louis, MO, USA), xylasine was purchased from Agener União (Calmiun^®^, São Paulo, Brazil) and ketamine was purchased from Holliday-Scott SA (Buenos Aires, Argentina). The extract and fractions were dissolved in saline solution (vehicle). All substances were prepared immediately before use, and the reagents used were of analytical grade. *Bothrops jararaca* venom was obtained from the Butantan Institute (São Paulo, Brazil). The venom was maintained at −80 °C until use, and venom solutions were prepared with phosphate-buffered saline (PBS). The proteins from the venom were quantified by the colorimetric method of Bradford [27], using bovine serum albumin. The amount of venom was expressed by its protein content (mg).

### 3.2. Dosage of Proteins

The concentration of proteins present in the venom of *Bothrops jararaca* and in the dermis of mice was determined by the colorimetric method of Bradford [27], using bovine serum albumin as a standard.

### 3.3. Plant

*Davilla elliptica* St. Hil. was collected (July 2002) in Monte do Carmo Tocantins, Brazil, by Clélia Akiko Hiruma-Lima. Solange de Fatima Lolis identified the species, and the voucher specimen was deposited in the Tocantins Federal University Herbarium as number 4583. The air-dried and powdered leaves (1.0 kg) of *Davilla elliptica* were extracted exhaustively with methanol (8 L) at room temperature for 1 week. The solvents were evaporated at 60 °C under reduced pressure, affording the methanolic extract of *Davilla elliptica* (yield of 18.7%). The methanol extract was stored at 5 °C until the experiments were conducted. Approximately 100.0 mg of the methanol extract was dissolved in 10.0 mL of methanol/H_2_O (2:8, *v*/*v*). For the removal of possible contaminants, the solution was purified by solid phase extraction (SPE), using Phenomenex Strata C_18_ cartridges (500 mg of stationary phase) previously activated with 5 mL of methanol and equilibrated with 5 mL of ultra-pure water. Tannins were eluted from the cartridges using 20 mL of a mixture of methanol/H_2_O (2:8, *v*/*v*), and flavonoids were eluted using 20 mL of methanol. Once again, the solvents were evaporated at 60 °C under reduced pressure and evaporated to dryness under a stream of nitrogen. Approximately 20 mg of the residue was redissolved in 2 mL of methanol and filtered through a 0.45-µm Teflon membrane filter. The final volume was adjusted to 5 mL with methanol/H_2_O (2:8, *v*/*v*), and aliquots of 20 µL were submitted to analysis by high-performance liquid chromatography coupled with a photodiode array detector (HPLC-PAD). The classes of secondary metabolites were identified by an analysis of the UV spectra. The HPLC-PAD qualitative analyses were performed using a Jasco liquid chromatography system equipped with a PU-2089 quaternary solvent delivery pump, an MD-2010 PAD and an AS-2055 autosampler. The analytical column was a Phenomenex Synergi Hydro RP18 (250 × 4.6 mm i.d.; 4 µm), and the elution system employed was the same one used in the identification of the polyphenolic compounds found in the infusion of the leaves of *Davilla elliptica* [11]. The procedure was repeated until sample masses of the tannin and flavonoid fractions large enough for the biological experiments could be obtained.

### 3.4. Animals

The experiments were conducted using male Swiss mice (weight, 25–35 g; approximately 6 weeks of age) and male Wistar rats (weight, 150–200 g; approximately 2 months of age) obtained from the Central Animal House (UNESP) in Botucatu, Brazil. All animals were housed in collective cages at a controlled temperature (23 ± 2 °C), under a 12-h light/dark cycle, with free access to food and water. All animal care and experimental procedures were performed in accordance with the ethical statements established by the National Guidelines for the Use of Experimental Animals of Brazil and conducted following the protocols approved on 8 August 2008 by the Committee for the Ethical Use of Animals (No.30/08-CEEA). The animals were habituated to the laboratory conditions for at least 1 h before testing, and all experiments were performed during the light phase of the cycle. All efforts were made to demonstrate consistent effects of the drug treatments and to minimize both the number of animals used and their suffering.

### 3.5. Carrageenan-Induced Hind Paw Oedema in Rats

Paw oedema was induced in male Wistar rats by carrageenan injection [28]. Groups of rats (*n* = 5–6) were treated via the intraperitoneal route with vehicle (1 mL/kg), methanolic extract from *D. elliptica* (7.81, 15.62 or 31.25 mg/kg), the fraction of tannins (15.62 mg/kg), the fraction of flavonoids (15.62 mg/kg) or piroxicam (30 mg/kg, by oral route) 1 h prior to carrageenan injection. To induce paw oedema, 0.1 mL of carrageenan (1% in sterile saline solution) was injected intradermally on the plantar side of the right hind paw. The rat paw volume was measured up to the ankle joint with a plethysmometer (Ugo Basile, Varese, Italy) at 1, 2, 3 and 4 h after the injection of carrageenan. The anti-inflammatory effects of the extract and fractions from *D. elliptica* were determined based on the decrease in the difference in paw oedema volume between the left (without carrageenan) and right (with carrageenan) hind paws (mL).

### 3.6. Intravital Microscopy

Male Swiss mice were divided into three groups of 11 animals each. After that, one group received the tannins fraction from *Davilla elliptica* (15.62 mg/kg) intraperitoneally, and 30 min later, all mice underwent surgery to expose the cremaster muscle [29]. The surgical preparation of the cremaster muscle was conducted as described previously [30]. The mice were anesthetized by an i.p. injection of 2% xylasine with 0.5 mg/kg of ketamine. The scrotum was opened, and the cremaster muscle was exteriorized. After a longitudinal incision with a cautery and the spreading of the muscle over a cover glass, the epididymis and testis were mobilized and pinned aside, leading to full microscopic access to the cremaster muscle microcirculation. The exposed tissue was superfused with 37 °C warmed bicarbonate-buffered saline, pH 7.4. The post-capillary venules, with diameters of 25–40 mm, were chosen, and the interaction of leukocytes with the luminal surface of the venular endothelium was evaluated by counting the number rolling 30 min after the application of lipopolysaccharides (LPS) (groups treated with vehicle and tannin fractions). Rolling leukocytes were defined as those moving at a velocity less than erythrocytes and demonstrating a clear rolling motion.

### 3.7. Gelatin Zymography for MMP-2 and MMP-9

The dermonecrosis assay of *Bothrops jararaca* and the quantification of the MMP activities extracted from the dermis of mice were based on the methods described by Saravia-Otten and colleagues [24], with modifications. To evaluate the effects of the extract and fractions from *Davilla elliptica* on the activity of MMPs, mice were divided into 9 groups (5 mice per group), as described below, that received intradermal injections:

G1: negative control, received PBS, 0.1 mL/animal; G2: Venom (35 µg/animal in 0.1 mL of PBS); G3: Venom + methanolic extract (35 µg of venom incubated with 700 µg of extract in 0.1 mL of PBS/animal); G4: Venom + tannins fraction (35 µg of venom incubated with 700 µg of tannins fraction in 0.1 mL of PBS/animal); G5: Venom + flavonoids fraction (35 µg of venom incubated with 700 µg of flavonoids fraction in 0.1 mL of PBS/animal); G6: 700 µg of tannins fraction in 0.1 mL of PBS/animal; G7: 700 µg of flavonoids fraction in 0.1 mL of PBS/animal; G8: 700 µg of methanolic extract in 0.1 mL of PBS/animal; and G9: 700 µg of methanolic extract in 0.1 mL of PBS/animal 1 h after venom (35 µg/animal in 0.1 mL of PBS). The groups G1, G6, G7 and G8 were used to determine the effects of the vehicle (PBS), the tannins fraction, the flavonoids fraction and the methanolic extract from *D. elliptica*, respectively. Groups G3, G4 and G5 received venom incubated with the extract, tannins or flavonoid fraction, and G9 received the extract after 1 h of venom administration. After 3 days, the animals were killed, and their dermises were collected and mechanically homogenized. Equal amounts of total protein (30 µg/lane) consisting of a pool of five animals per group (6 µg/animal) were subjected to electrophoresis in gelatin-containing SDS-PAGE gels (8%) under non-reducing conditions [20]. After electrophoresis, the gels were washed in Triton X-100 (2.5%) twice for 15 min and then in Tris-HCl (50 mM, pH 8.4) twice for 5 min. Subsequently, the gels were incubated for 18 h overnight at 37 °C in Tris-HCl (50 mM, pH 8.4) containing 5 mM CaCl_2_ and 1 μM ZnCl_2_. After incubation, the gels were stained with Coomassie Brilliant Blue (0.25%). The areas of proteolysis were evident as clear bands against a dark blue background. The gelatinolytic activity of the MMPs was analysed by obtaining the integrated optical density (IOD) of the bands using Image Master VDS, version 3.0, coupled with the Image Master VDS apparatus (Amersham Pharmacia Biotech Del, Milwaukee, WI, USA). The results are presented as fold increases (fold increase = treatments IOD/PBS group IOD).

### 3.8. Statistical Analyses

The results are expressed as the mean ± standard error of the mean (SEM), and the differences between the groups were determined by analysis of variance (ANOVA). Significant differences were analysed by the Dunnett’s post-test (for three or more groups) with *p* < 0.05 considered significant. For the zymography assay, the results were analysed in a descriptive manner according to Saravia-Otten and colleagues [24].

## 4. Conclusions

The results of the present study demonstrate that the methanolic extract and the tannins fraction from *D. elliptica* have anti-inflammatory effects, and both were able to attenuate oedema and the rolling of leukocytes as well as to decrease the activity of MMP-9. All of these results will be useful for developing anti-inflammatory adjuvants in the treatment of tissue injury in accidents by *Bothrops*.
